# Dichloroacetate and PX-478 exhibit strong synergistic effects in a various number of cancer cell lines

**DOI:** 10.1186/s12885-021-08186-9

**Published:** 2021-04-30

**Authors:** Jonas Parczyk, Jérôme Ruhnau, Carsten Pelz, Max Schilling, Hao Wu, Nicole Nadine Piaskowski, Britta Eickholt, Hartmut Kühn, Kerstin Danker, Andreas Klein

**Affiliations:** Charité – Universitätsmedizin Berlin, Corporate Member of Freie Universität Berlin, Humboldt-Universität zu Berlin, and Berlin Institute of Health, Charitéplatz 1, 10117 Berlin, Germany

**Keywords:** PX-478, HIF-1α inhibition, Dichloroacetate, Synergism, Cancer therapy, Drug combination, Cancer cell lines, Metabolism

## Abstract

**Background:**

One key approach for anticancer therapy is drug combination. Drug combinations can help reduce doses and thereby decrease side effects. Furthermore, the likelihood of drug resistance is reduced. Distinct alterations in tumor metabolism have been described in past decades, but metabolism has yet to be targeted in clinical cancer therapy. Recently, we found evidence for synergism between dichloroacetate (DCA), a pyruvate dehydrogenase kinase inhibitor, and the HIF-1α inhibitor PX-478. In this study, we aimed to analyse this synergism in cell lines of different cancer types and to identify the underlying biochemical mechanisms.

**Methods:**

The dose-dependent antiproliferative effects of the single drugs and their combination were assessed using SRB assays. FACS, Western blot and HPLC analyses were performed to investigate changes in reactive oxygen species levels, apoptosis and the cell cycle. Additionally, real-time metabolic analyses (Seahorse) were performed with DCA-treated MCF-7 cells.

**Results:**

The combination of DCA and PX-478 produced synergistic effects in all eight cancer cell lines tested, including colorectal, lung, breast, cervical, liver and brain cancer. Reactive oxygen species generation and apoptosis played important roles in this synergism. Furthermore, cell proliferation was inhibited by the combination treatment.

**Conclusions:**

Here, we found that these tumor metabolism-targeting compounds exhibited a potent synergism across all tested cancer cell lines. Thus, we highly recommend the combination of these two compounds for progression to in vivo translational and clinical trials.

**Supplementary Information:**

The online version contains supplementary material available at 10.1186/s12885-021-08186-9.

## Introduction

In the last decade, combinatorial approaches for cancer therapy have become increasingly popular [1]. Drugs designed to act against individual molecular targets can hardly combat a multigenic disease such as cancer [2]. However, synergistic drug combinations can lead to reduced drug doses with less pronounced side effects, increased response rates and attenuated likelihoods of drug resistance [1–3].

In a previous work [4], we screened 14 selected compounds, including dichloroacetate (DCA) and PX-478, for synergistic interactions in cancer cell lines. The combination of DCA and PX-478 displayed significantly stronger effects on cell viability than either single compound. Therefore, we aimed to further investigate this combination using a widely accepted method of quantifying synergism over the whole dose-response curve introduced by Chou and Talalay [5].

### Compounds

DCA, a chlorinated carboxylic acid that was originally administered in the treatment of hereditary lactate acidosis [6], is an inhibitor of pyruvate dehydrogenase kinase (PDK). Thus, it leads to increased pyruvate dehydrogenase activity and therefore to an increase in pyruvate decarboxylation to acetyl-CoA, partially reversing the Warburg effect [7]. The Warburg effect describes alterations in tumor metabolism that lead to enhanced aerobic glycolysis and a reduction in oxidative phosphorylation. These alterations, while being less energy efficient, provide the necessary building blocks the tumor needs for proliferation [8, 9]. Furthermore, the reduction in cell respiration results in suppression of the mitochondrial-K^+^ channel axis and thus hyperpolarisation of the mitochondrial membrane. Consequently, the release of cytochrome c and AIF is impaired, leading to apoptosis resistance [10]. DCA was found to normalise this axis and thereby induce the apoptosis of cancer cells [11, 12]. In addition to its effects on the mitochondrial membrane potential, DCA is believed to lead to a significant increase in reactive oxygen species (ROS) generation, which plays an important role in the induction of apoptosis [13–17]. In contrast, other authors reported that DCA may function as a sensitiser for ROS-induced alterations but did not significantly increase ROS production per se [16, 18]. In addition, DCA has been shown to positively regulate p53 as well as to downregulate autophagy, thereby leading to enhanced tumor cell apoptosis and attenuated cell proliferation [19, 20].

PX-478 is a small molecule that interferes with the transcription and translation of hypoxia-inducible factor-1α (HIF-1α) and leads to diminished deubiquitination of HIF-1α [21]. HIF-1α is physiologically activated by hypoxia and mediates multiple cellular alterations via transactivation of various target genes, such as GLUT1, LDHA and VEGF, and hence increases aerobic glycolysis in order for the cell to sustain hypoxic conditions [22]. Hence, PX-478-mediated inhibition of HIF-1α was found to induce apoptosis and cell cycle arrest in cancer cells [23, 24]. In oesophageal squamous cell cancer, PX-478 induces apoptosis, reduces cell proliferation and inhibits epithelial-mesenchymal transition [25]. Welsh et al. identified that the antitumor effect of PX-478 is positively correlated with HIF-1α levels in human xenografts [26]. In a study by Lang et al., PX-478 acted synergistically with an ROS inducer, ATO, leading to more efficient ROS-induced apoptosis via blocking ROS clearance by the HIF-1/FOXO1/SESN3 pathway [24].

HIF-1α-mediated inhibition of mitochondrial ROS production (as a reaction to ROS accumulation, hypoxia and cytokine stimulation) is achieved partially through a decrease in the production of acetyl-CoA via upregulation of PDK-1 and -3, the direct targets of DCA [27, 28]. Additionally, DCA-mediated inhibition of PDK leads to HIF-1α inhibition and, thereby supresses angiogenesis [14].

Apart from preliminary results indicating a likely synergism [4], the anticipated interplay of DCA and PX-478 regarding ROS generation, apoptosis and proliferation makes this combination especially interesting for further investigations.

In this study, we examined the effects of the combination of DCA and PX-478 on eight cancer cell lines and the non-cancerous cell line HEK-293. In addition, we studied the impact of the combination on ROS generation, apoptosis induction and cell cycle arrest.

## Methods

### Cell culture

The breast cancer cell lines MCF-7 and MDA MB-231 were a kind gift from Göran Landberg (Sahlgrenska Cancer Center, University of Gothenburg, Gothenburg, Sweden). The colon cancer cell line HT-29, the hepatocellular cancer cell line HEPG2, the cervical cancer cell line HeLa and the adenocarcinoma lung cancer cell lines A549 and H441, as well as the non-cancerous cell line HEK-293, were purchased from the American Type Culture Collection (ATCC). The glioblastoma cell line U251 was a kind gift from Kai Murk (Charité Berlin, Germany). A549, HEK-293, HeLa, HEPG2, HT-29, MCF-7 and U251 cells were cultured in DMEM, and H441 and MDA-MB-231 cells were cultured in DMEM/F12. All media contained penicillin/streptomycin (100 U ml^− 1^), L-glutamine (DMEM: 584 mg l^− 1^, DMEM/F12: 365.1 mg l^− 1^) and 10% heat-inactivated foetal calf serum (PAN Biotech, Germany). The humidified incubator was set at 37 °C with 5% CO_2_. Cells were harvested using 0.05% trypsin/0.02% ethylenediaminetetraacetic acid (EDTA) in PBS.

### Compounds

PX-478 (Hölzel Diagnostika Handels GmbH, Cologne, Germany) and DCA (Sigma-Aldrich, Munich, Germany) were dissolved in distilled water.

### Cell viability and cell proliferation assays

A total of 0.75 × 10^4^ A549, 1 × 10^4^ HEK-293, 0.3 × 10^4^ HeLa, 0.6 × 10^4^ HEPG2, 1.5 × 10^4^ HT-29, 0.5 × 10^4^ MCF-7, 1.5 × 10^4^ MDA-MB-231, 1 × 10^4^ H441 and 0.3 × 10^4^ U251 cells per well were seeded in flat bottom 96-well plates. After 24 h, when the cells were approximately 50% confluent, DCA, PX-478 or the combination was added. After 48 h of further incubation, a sulforhodamine B (SRB) assay was performed. For the SRB assay, cells were fixed with 10% trichloroacetic acid (w/v) and stained with 0.06% SRB in 1% acetic acid for 30 min. Cells were then repeatedly washed with 1% acetic acid (v/v) and dissolved in 10 mM Tris (pH 10.5). The protein mass was measured by determining the optical density at a wavelength of 492 nm in a microplate reader. Additionally, in HT-29 cell MTT assays were performed according to the manufacture’s instructions (data are shown in additional file 1). All experiments were performed independently three times with at least 2 technical triplicates (mostly with 3).

Dose-response curves were generated using GraphPad Prism 7.05 statistical analysis software. The half-maximal effective concentration (EC_50_) of each compound was determined via nonlinear regression.

### Confirmation of synergism

Synergism was evaluated with four to seven different concentrations (mostly with 6), as suggested by Chou and Talalay [5].

Cells were treated with the combination of DCA and PX-478 at a constant EC_50_:EC_50_ ratio as well as with the single compounds alone. Significant differences between each single compound and the combination were assessed by an unpaired t-test. Only concentrations with *p*-values of ≤0.05 for both single compounds compared to the combination were considered to exhibit significant differences and are marked with an asterisk (*) in the figures.

Combination indices (CIs) were calculated using CompuSyn software [29]. The CI is a quantitative value indicating the synergism of a drug combination at specific concentrations. A value of less than 0.9 indicates synergism (the lower the CI, the stronger the synergism). Values from 0.9 to 1 indicate a nearly additive effect, and a CI value of greater than 1.1 indicates antagonism [30]. CI values were calculated as follows:

$$ CI=\frac{(D)_1}{(Dx)_1}+\frac{(D)_2}{(Dx)_2} $$

In the numerators, (D)_1_ and (D)_2_ are the concentrations of drug 1 and drug 2, respectively, in the drug combination that have a certain effect on cell viability (x %). In the denominators, (Dx)_1_ and (Dx)_2_ are the concentrations of each drug alone (drug 1 or drug 2, respectively) that are necessary to obtain the same effect (x %) as the drug combination (both drug 1 and drug 2). The concentrations (Dx)_1_ and (Dx)_2_ were calculated by CompuSyn with reference to the cell viability data for the respective compounds. To enhance analytical robustness, most concentrations of the compounds were doubled. Therefore, potential calculation errors were minimised, as suggested by Zhao et al. [31]. To generate the median-effect plots, the following equation was used:

$$ {D}_x={D}_m{\left[\frac{fa}{1- fa}\right]}^{1/m} $$

where Dm is the median effective dose, m is the slope of the median-effect curve, and fa is the fraction affected. Since calculation of a CI value is appropriate only when neither single compound has an effect close to 100%, the respective CI values are not shown in the Results section [31]. All data collected in this study can be found in additional file 1 (additional file 1).

### Membrane lipid oxidation rate

HT-29 cells were seeded in 10 cm diameter Petri dishes and treated with the EC_50_ dose of DCA, the EC_50_ dose of PX-478 or the combination after 24 h when the cells were approximately 80% confluent. After incubation for an additional 48 h, cells were harvested with trypsin, pelleted and resuspended in 500 μl of PBS. For lipid extraction, cells were homogenised in a mixture of methanol:chloroform:water (2:1:1 by volume) using a modified Bligh/Dyer method. The extracted lipid suspension was bubbled with argon to prevent artificial oxidation. Then, alkaline hydrolysis was carried out, and the resulting free fatty acids were analysed by reversed-phase HPLC (RP-HPLC). Arachidonic acid and its oxygenated derivative 10−/15-hydroxyeicosatetraenoic acid (HETE) were identified by their specific retention times and UV spectra and were quantified via integration [32].

### Flow cytometric analysis

Samples were analysed with BD FACS Calibur and Cell Quest.

#### Detection of intracellular ROS

Intracellular ROS were detected via an oxidation-sensitive fluorescent probe (2′,7′-dichlorodihydrofluorescein diacetate [H2DCFDA], Bio-Techne GmbH, Germany). HeLa and MCF-7 cells were seeded in 6 cm diameter Petri dishes and treated after 24 h at a confluence of 50%. Cells were treated with the EC_50_ dose of DCA, PX-478 or the combination for 48 h. Then, cells were harvested and washed twice with PBS. Next, the cells were incubated with 50 μM H2DCFDA at 37 °C for 20 min in the dark and were then placed on ice. Cells were washed 2 more times before being analysed by flow cytometry.

#### Evaluation of apoptosis by Annexin-V-FITC and propidium iodide staining

HeLa and MCF-7 cells were seeded in 6 cm diameter Petri dishes and incubated for 24 h to a confluence of approximately 60%. After 24 h, cells were treated with PX-478, DCA or the combination and harvested 48 h later. The following concentrations were used: HeLa cells—EC_50_ DCA and 0.5 x EC_50_ PX-478; MCF-7 cells—EC_50_ DCA and EC_50_ PX-478. Cells were washed twice with PBS, placed on ice immediately, transferred to binding buffer (10 mM Hepes, 140 mM NaCl, 2.5 mM CaCl_2_; pH 7.4) and stained with Annexin-V-FITC (Hölzel Diagnostika Handels GmbH, Germany) in the dark according to the manufacturer’s instructions. After 15 min, propidium iodide (50 μg/ml) was added, and the cells were analysed by flow cytometry.

### Western blot analysis

For Western blotting, cells were seeded in 6 cm diameter Petri dishes, grown to approximately 80% confluence and treated with the noted compounds. 24 h later, the cells were washed with PBS and lysed with lysis buffer (50 mM β-glycerophosphate pH 7.6, 1.5 mM EGTA, 1.0 mM EDTA, 1% (v/v) Triton X-100, 0.2% (v/v) protease inhibitor cocktail, 0.4% (v/v) PMSF, 100 mM sodium vanadate, 500 mM NaF). The samples were separated under reducing conditions by 10% SDS-PAGE and transferred to nitrocellulose membranes (Thermo Fisher, Rockford, USA). The primary antibodies and the corresponding working concentrations are listed in Table 1. Proteins were detected using SuperSignal West Pico Chemiluminescent Substrate (Pierce, Thermo Fisher Scientific, Bonn, Germany). Signals were visualised using a VersaDoc™ 4000 MP and QuantityOne® 4.6.5 software (BioRad Laboratories, Munich, Germany) and quantified using ImageJ 1.52a software (National Institute of Health, USA; version 1.8.0_112).

**Table 1:** List of antibodies

Antibody raised against	Purchased from	Source	Dilution
β-actin	Cell Signaling (Danvers, USA)	Mouse	1:4000
PARP/cleaved PARP (9542)	Cell Signaling (Danvers, USA)	Rabbit	1:1000
Retinoblastoma p795 (9301)	Cell Signaling (Danvers, USA)	Rabbit	1:1000
Cyclin D1 (DCS-6)	Thermo Scientific (Waltham, USA)	Mouse	1:200

### Metabolic assays

MCF-7 cells were seeded in an XF 96-well culture microplate (Agilent, Santa Clara, USA) at 3 × 10^4^ cells per well in 180 μl of prewarmed assay medium. After 24 h, a mitochondrial respiration assay or glycolytic rate assay was performed with a Seahorse XFe96 Analyzer (Agilent Technologies). For the mitochondrial respiration assay, the oxygen consumption rate (OCR) was measured using the mitochondrial stress test procedure in XF media (nonbuffered DMEM containing 10 mm glucose, 2 mm L-glutamine and 1 mm sodium pyruvate). The glycolytic rate was measured in accordance with the Agilent Seahorse XFp Glycolytic Stress Test Kit instructions. After four measurements of either the baseline OCR or baseline extracellular acidification rate (ECAR), DCA solution was injected into the appropriate wells to the desired working concentration. Before each measurement, the assay medium was gently mixed to restore normal oxygen tension and pH in the microenvironment surrounding the cells. Two hours after treatment with DCA (6 measurements), the actual mitochondrial respiration assay or glycolytic stress test was performed. When metabolic analysis was complete, the cells were immediately fixed, and an SRB assay was performed as described above for data normalisation. Graphs were produced using GraphPad Prism 7.05 statistical analysis software. Glycolytic capacity and maximal respiration (Fig. 5) were calculated as follows:

- maximal respiration (OCR) = (maximum rate measured after injection of carbonyl cyanide-4-(trifluoromethoxy)phenylhydrazone [FCCP]) – (non-mitochondrial respiration rate)
- non-mitochondrial respiration (OCR) = minimum rate measured after injection of rotenone & antimycin A)
- Glycolytic capacity (ECAR) = (maximum rate measured after injection of oligomycin) – (non-glycolytic acidification rate)
- non-glycolytic acidification (ECAR) = minimum rate measured after injection of 2-deoxy-D-glucose (2DG).

### Statistical analysis

Statistical analysis was performed using unpaired T-tests in GraphPad Prism 7.05 statistical analysis software. Differences with a *p*-value of ≤0.05 were considered significant: significant differences compared to the control are marked with an asterisk (*), while significant differences between the combination and both the control and each single compound are marked with two asterisks (**). All experiments were performed with at least 2 technical and 3 biological replicates.

## Results

### The combination of DCA and PX-478 produces synergistic effects in eight cancer cell lines and shows only minimal effects on the non-cancerous cell line HEK-293

**Fig. 1:**
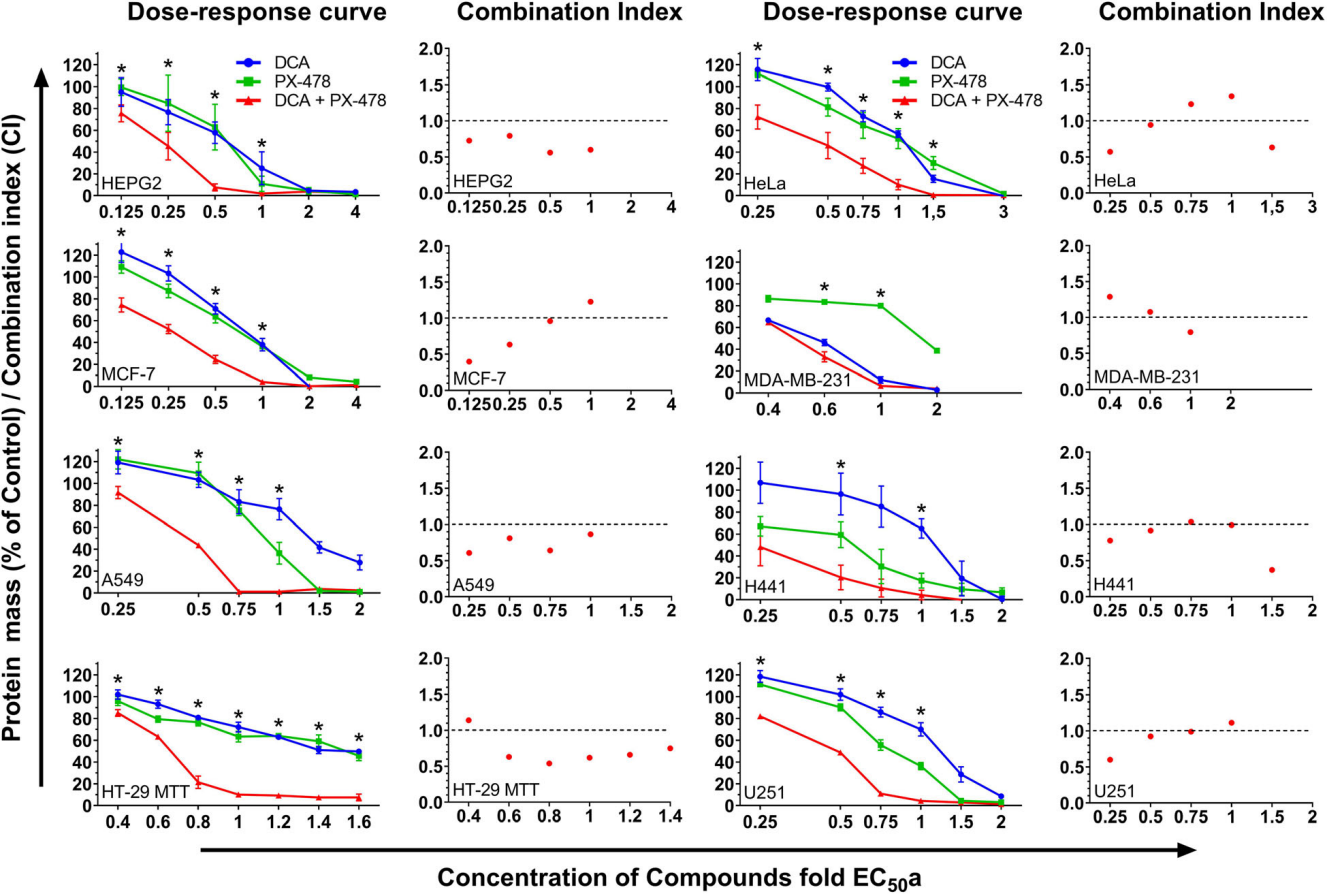
Synergistic interactions between DCA and PX-478 in eight cancer cell lines. Figure 1 shows the dose-response curves for DCA, PX-478 and their combination in eight different cell lines as well as the respective CIs (shown to the right of each dose-response curve). Cells were seeded in 96-well plates and treated at a confluence of approximately 50%. Forty-eight hours later, an SRB (protein mass) assay was performed. If applicable, a CI was calculated with CompuSyn for each concentration. A CI of less than 0.9 indicates synergism, a CI between 0.9 and 1.1 indicates a nearly additive effect, and a CI of greater than 1.1 indicates antagonism. Approximated EC_50_ values were used (EC_50_a) at a constant EC_50_a:EC_50_a ratio. Concentrations for which the effect of combination was significantly different from that of both single compounds and the control (*p* ≤ 0.05, unpaired T-Test) are marked with an asterisk (*). Synergistic interactions were confirmed for all cancer cell lines, as indicated by the CI values and predominant left shifts of the curves. Without exception, the effects of the drug combination surpassed the effects of each single compound

In this study, we evaluated the effects of DCA and PX-478 on eight cancer cell lines, including lung (A549 and H441), breast (MCF-7 and MDA-MB-231), cervical (HeLa), hepatocellular (HepG2), colon cancer (HT-29) and glioblastoma (U251) cell lines (Fig. 1). The EC_50_ values used for treatment in the combinatorial experiments were determined for all cell lines in preceding experiments and are henceforth referred to as the approximated half-maximal effective concentration (EC_50_a) values [4] (see additional file 1). The actual EC_50_ values for the experiments conducted herein were calculated afterwards (see Table 2).

**Table 2:** EC_50_ values for the single compounds and the combination

Cell line	EC_**50**_ DCA (mM)	EC_**50**_ PX-478 (μM)	EC_**50**_ Combination DCA (mM)/PX-478 (μM)	Best CI
**A549 (lung)**	41.9	30	14.2/15.8	0.61
**H441 (lung)**	38.6	23.5	8.3/11.9	0.78
**HeLa (cervical)**	21.2	13.4	8.9/5.8	0.57
**HEPG2 (hepatocellular)**	21.4	17.7	8.4/6.3	0.56
**HT-29 (colon)**	26.5	75.6	18.1/28.2	0,65
**MCF-7 (breast)**	31.5	11.2	10.2/4	0.4
**MDA-MB-231 (breast)**	26.1	276	23.4/79	0.8
**U251 (glioblastoma)**	25	30.5	9.5/16.8	0.6

While the combination showed synergistic effects in six investigated tumor entities, the combination exhibited synergistic effects over the complete dose-response curve in A549 (lung adenocarcinoma) and HEPG2 (hepatocellular carcinoma) cells with CI values ranging from 0.61 to 0.87 and 0.56 to 0.79, respectively.

EC_50_ data and best CI values are listed in Table 2. As illustrated, the synergism between DCA and PX-478 was observed in all analysed cell lines, with the lowest CI value in MCF-7 at 0.125 x EC_50_ (CI = 0.4). To minimise extrapolation errors, we calculated CI values relying on experimental data and eliminated CI values for concentrations where the effect of either single compound was too close to 100%, as suggested by Zhao et al. [31]. Interestingly, the combination of DCA and PX-478 strongly affected cell viability or the protein mass in all cell lines, leading to a left shift in the dose-response curves. The combination treatment allowed the concentration of each single drug to be noticeably reduced (Table 2). For example, in MCF-7 cells, the EC_50_ values of DCA and PX-478 were reduced by 68 and 64%, respectively. Collectively considering all cell lines, the EC_50_ values of the compounds were profoundly reduced by an average of 60.7% when used in combination relative to when used as single agents.

Comparison of the EC_50_ values of PX-478 in HT-29 and MDA-MB-231 cells indicates that noticeably higher doses were needed in these cell lines than in the other cell lines, indicating resistance to PX-478. For MDA-MB-231 cells, the resistance to PX-478 resulted in the highest CI value compared to the other cell lines. Interestingly, the dose-response curve for DCA was close to that for the combination (Table 2 and Fig. 1). However, a stronger synergism was shown for HT-29 cells even though a higher dose of PX-478 was required (CI = 0.65).

**Fig. 2:**
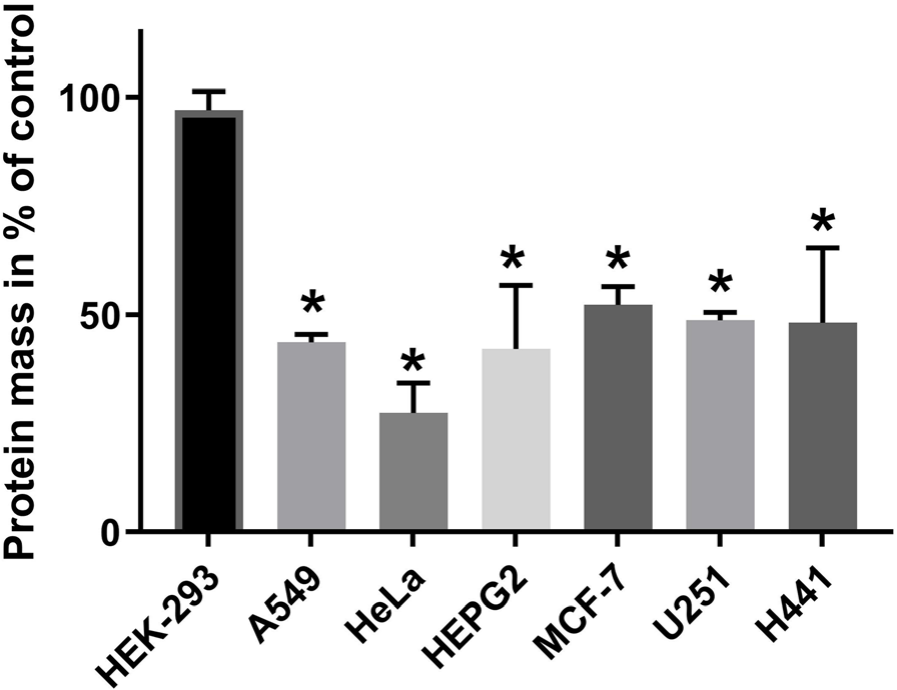
The combination of DCA and PX-478 shows significantly lower effects on the non-cancerous cell line HEK-293 than on the six PX-478-sensitive cancer cell lines (A549, HeLa, HEPG2, MCF-7, U251 and H441). HEK-293 cells were seeded in 96-well plates and treated with 15 mM DCA and 15 μM PX-478 or the combination at a confluence of approximately 50%. Forty-eight hours later, an SRB assay was performed. The combination had no significant effect (97% protein mass) compared to the control (*p* = 0.3). The effect of the combination of 15 mM DCA and 15 μM PX-478 on HEK-293 cells was compared to the effect of similar or lower concentrations of the combination on the tested cancer cell lines (A549: 15 mM and 16.5 μM, HeLa: 15.5 mM and 10 μM, HEPG2: 10.5 mM and 8 μM, MCF-7: 10 mM and 4 μM, U251: 10 mM and 18.5 μM and H-441: 9 mM and 12.5 μM respectively). Data points used to generate the dose-response curves in Fig. 1 were used for comparison. The bars are marked with an asterisk when the effect on a cancer cell line was significantly stronger than that on HEK-293 cells. All six PX-478-sensitive cancer cell lines were significantly more sensitive than HEK-293 cells to the combination of DCA and PX-478. HT-29 and MDA-MB-231 cells were not compared, since due to the described resistance, no doses of PX-478 close to 15 μM were used. This figure is not applicable for comparing effects between the different cancer cell lines, since different concentrations of the compounds were used. For comparisons of synergism, please see Fig. 1 and Table 2

The six cell lines that were sensitive to PX-478 were significantly more sensitive to the combination of DCA and PX-478 than the immortalised non-cancerous cell line HEK-293 at a comparable concentration (Fig. 2). For example, in MCF-7 cells, 10 mM DCA and 4 μM PX-478 led to a reduction of 48% in the protein mass, while 15 mM DCA and 15 μM PX-478 led to a reduction of only 3% in HEK-293 cells (*p* = 0.000007). Since we detected a PX-478 resistance in MDA MB-231 and HT-29 cells, we did not use concentrations of PX-478 in comparable dosages for the combination.

Table 2 lists the EC_50_ values for DCA, PX-478 and the combination of both in all tested cell lines. The EC_50_ values were calculated via curve fitting with the program GraphPad Prism. In the last column, the lowest CI value indicating synergism (CI < 0.9) is listed.

### The combination of DCA and PX-478 increases ROS levels and leads to apoptosis and cell cycle arrest

The existing data for PX-478 and DCA suggest some theories concerning the mechanisms underlying their synergism. In the following experiments, the effects of this combination on increasing reactive oxygen species generation, arresting the cell cycle and inducing apoptosis were investigated.

#### The combination of DCA and PX-478 increases ROS levels in HT-29, MCF-7 and HeLa cell lines

**Fig. 3:**
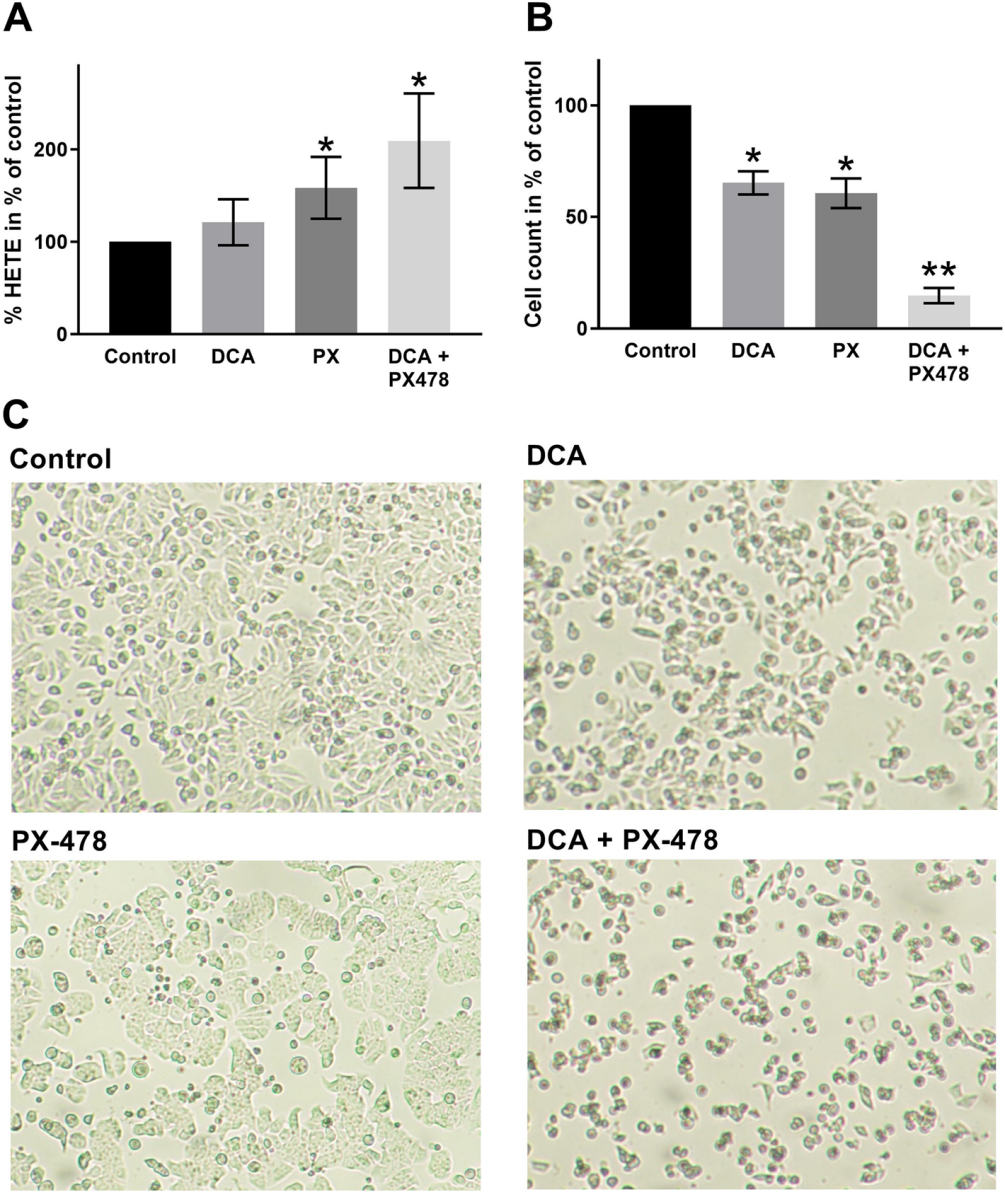
The combination of DCA and PX-478 leads to increased ROS activity in HT-29 cells. Figure 3 shows HPLC analysis results and cell counts for HT-29 cells. **a**: HPLC results for the proportion of arachidonic acid to its oxygenated derivatives 10−/15-HETE for drug treatment compared to the control treatment are presented. Cells were treated with either the EC_50_ dose of DCA, the EC_50_ dose of PX-478 or the combination. Cells treated with PX-478 alone and with the combination of DCA and PX-478 showed a significant increase in the oxidation ratio compared to that in control cells, although the difference between the combination and PX-478 was noticeable but not significant. **b**: The cell count as a percentage of the control cell count is presented. Treatment with the single compounds DCA and PX-478 led to significant reductions of 65 and 61%, respectively. Only 15% of the control cells remained after the combination treatment. Significant differences from the control are marked with an asterisk (*), while significant differences from both the control and each single compound are marked with two asterisks (**). **c**: The profound effects of DCA, PX-478 and DCA + PX-478 on cell confluency are shown. All cells were imaged at 40× magnification with a Nikon D90

To investigate the relevance of the combination to ROS production, we performed HPLC measurements with HT-29 cells to analyse the oxidation of arachidonic acid derivatives (Fig. 3a). DCA-treated cells showed a non-significant (21%, *p* = 0.21) increase in the 5- and 10-HETE levels compared to those in control cells. In cells treated with PX-478, the oxidation ratio was significantly increased by 58% compared to that in control cells (*p* = 0.04). The combination treatment led to a 109% increase in the oxidation ratio, which was significantly higher than that observed for the control treatment (*p* = 0.02) but did not differ significantly from that observed for PX-478 alone (*p* = 0.22).

**Fig. 4:**
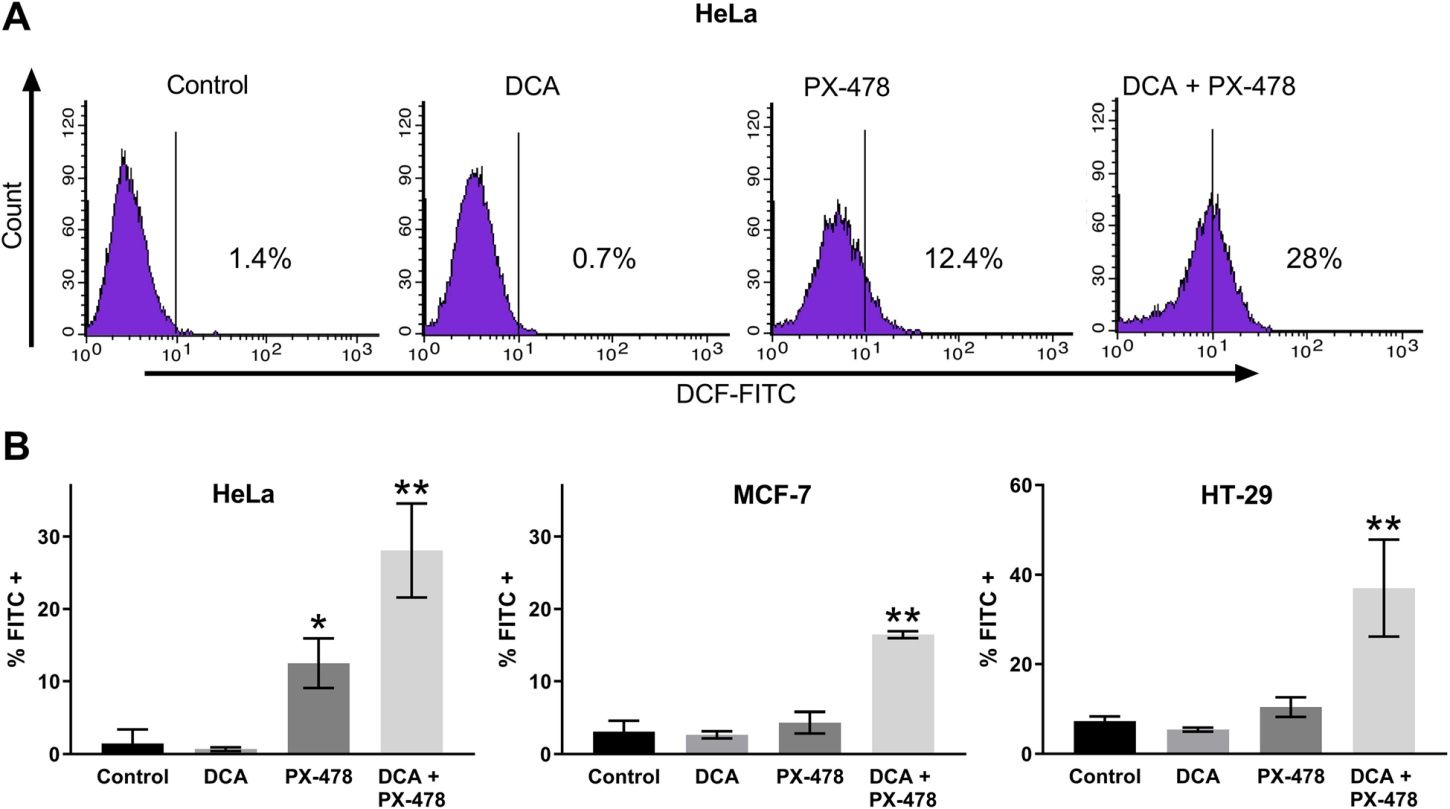
The combination of DCA and PX-478 leads to increased ROS activity. Figure 3 shows the ROS activity assessed by flow cytometric analysis with H2DCFDA in HeLa, MCF-7 and HT-29 cells. Cells were incubated to a confluence of 50% and were then treated with either DCA, PX-478 or the combination for 48 h. The following concentrations of the compounds were used: HeLa—DCA and PX-478, EC_50_; MCF-7 and HT-29—DCA, EC_50_ and PX-478, 0.5 x EC_50_. Panel **a** shows representative histograms for the analysis of DCF-FITC in HeLa cells. Panel **b** shows the results in HeLa, MCF-7 and HT-29 cells as bar graphs. Three independent experiments were performed. Significant results compared to the control are marked with an asterisk (*). The combination of DCA and PX-478 led to the highest ROS activity in all cell lines, which was significantly increased compared to that in the corresponding control, DCA-treated and PX-478-treated cells (marked with two asterisks [**])

Furthermore, we evaluated the relevance of this combination to ROS via FACS analysis with H2DCFDA in HeLa, MCF-7 and HT-29 cells (Fig. 4). H2DCFDA reacts with ROS, and fluorescent dichlorofluorescein (DCF) can be measured in the FL1 channel. The results shown in Fig. 4b confirmed our HPLC results in HT-29 cells. FACS analysis showed that compared to the control treatment, DCA did not affect ROS activity in any cell line. ROS production was significantly increased in HeLa cells (2 to 12%, *p* = 0.008) but not in MCF-7 cells (3 to 4%, *p* = 0.37) or HT-29 cells (7 to 10%, *p* = 0.089) treated with PX-478 alone compared to control cells. Compared to the single compounds, the combination led to significant increases of 28% (*p* = 0.021), 16% (*p* = 0.0002) and 37% (*p* = 0.014) in HeLa, MCF-7 and HT-29 cells, respectively. Thus, as our results in HeLa, MCF-7 and HT-29 cells suggest, increased ROS is likely to play an important role in the synergism of DCA + PX-478 combination treatment.

#### The combination of DCA and PX-478 leads to apoptosis and a reduction in proliferation

**Fig. 5:**
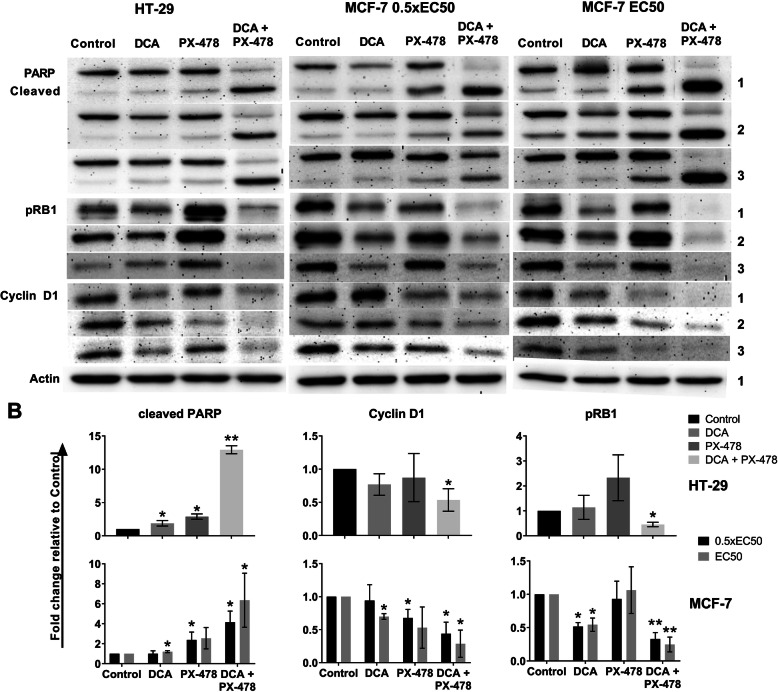
The combination of DCA and PX-478 leads to increased levels of cleaved PARP and reduced levels of Cyclin D1 and pRB1 in HT-29 and MCF-7 cells. Cells were incubated to a confluence of approximately 80% and treated with DCA and PX-478 at the following concentrations: HT-29—DCA, EC_50_ and PX-478, 0.5 x EC_50_; MCF-7—either DCA and PX-478, 0.5 x EC_50_ or EC_50_. Twenty-four hours later, the cells were harvested, and Western blot analyses were performed. **a**: Three independent Western blots are shown for each antibody except for β-actin (only one representative blot is shown here). The blots presented here are cropped; please see additional file 2 for full-length blots. **b**: The results are presented as the fold change relative to the control. Significant differences are marked with an asterisk (*). The level of cleaved PARP was significantly increased in HT-29 cells treated with the combination (*p* = 0.00002) compared to HT-29 cells treated with the single compounds. For MCF-7 cells, a clear trend was visible for both concentrations (*p* = 0.086 or *p* = 0.087, respectively). Significant differences compared to the control are marked with an asterisk (*); significant differences between the combination of DCA and PX-478 and each single compound are marked with two asterisks (**). The combination of DCA and PX-478 significantly reduced cyclin D1 and pRB1 levels in both cell lines and at both concentrations in MCF-7 cells

**Fig. 6:**
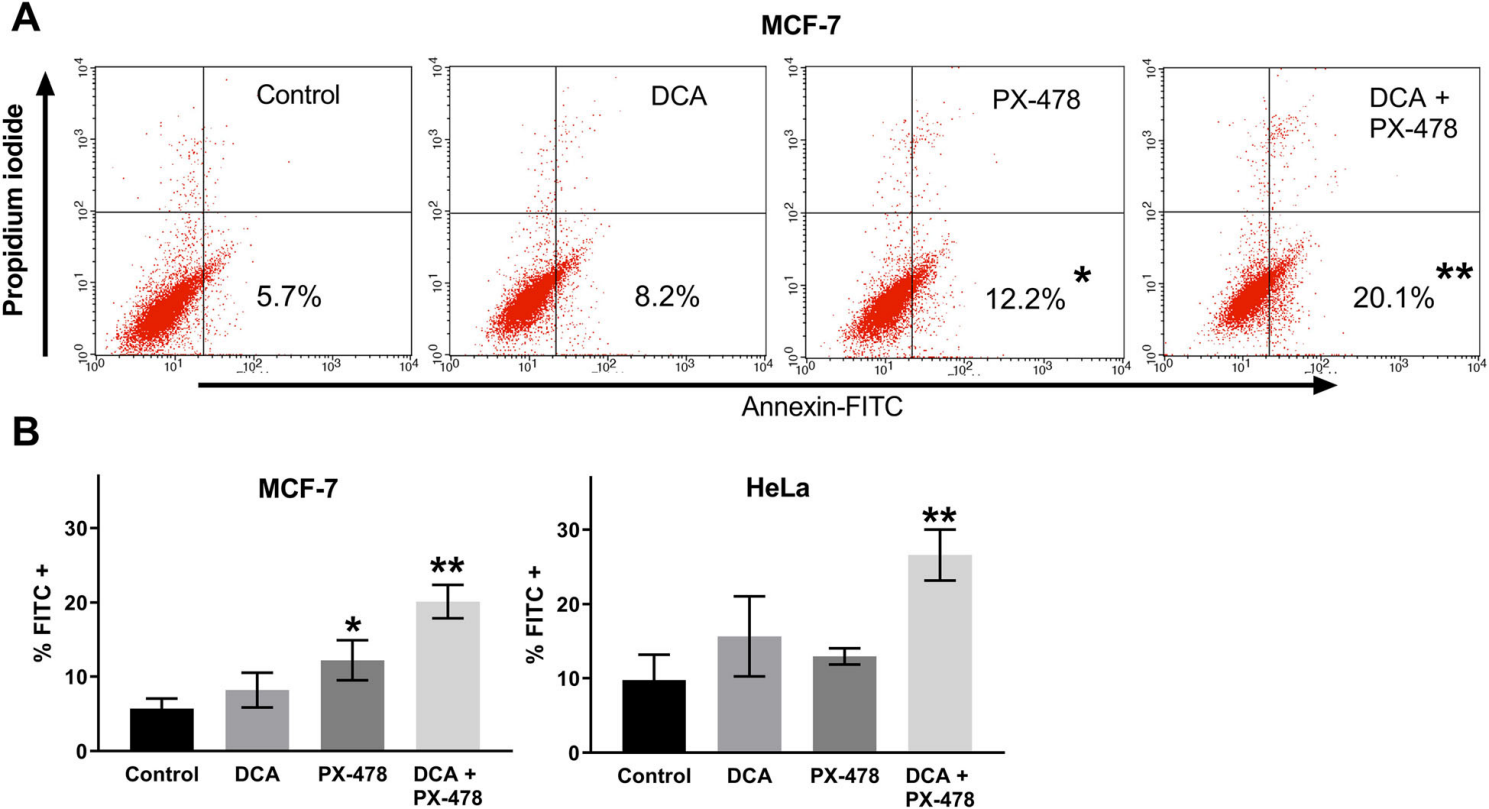
DCA + PX-478 induced significantly higher levels of apoptosis than DCA or PX-478 alone in MCF-7 and HeLa cells. Figure 6 shows the results of flow cytometric analysis with Annexin-V-FITC and propidium iodide in MCF-7 and HeLa cells. At 60% confluence, MCF-7 cells were treated with either the EC_50_ dose of DCA or the EC_50_ dose of PX-478, while HeLa cells were treated with the EC_50_ dose of DCA and 0.5 times the EC_50_ dose of PX-478. Three independent experiments were performed. Panel **b** shows bar graphs indicating the percentages of Annexin-VF-ITC-positive MCF-7 and HeLa cells. Significant differences compared to the control are marked with an asterisk (*). Representative dot plots are shown for MCF-7 cells in panel **a**. The combination of DCA and PX-478 led to the greatest percentage of Annexin-V-FITC-positive cells, and the percentage was significantly different from that of control, DCA-treated and PX-478-treated cells for both cell lines (**) (*p* = 0.004 and 0.042 for MCF-7 cells and HeLa cells, respectively)

Western blot analyses of PARP/cleaved PARP, Ser795-phosphorylated Retinoblastoma protein (pRB1) and Cyclin D1 were performed in HT-29 and MCF-7 cells (Fig. 5). In MCF-7 cells, two concentrations of DCA and PX-478 (EC_50_ and 0.5 x EC_50_) and the respective combinations were analysed. In HT-29 cells (DCA EC_50_ and PX-478 0.5 x EC_50_), the level of cleaved PARP was significantly higher in cells treated with the combination than in cells treated with the single compounds (*p* = 0.002). In MCF-7 cells, the combination led to the highest levels of cleaved PARP at both doses, with significant differences compared to control and DCA-treated cells but non-significant differences compared to PX-478-treated cells (*p* = 0.086 and *p* = 0.087). However, via FACS analysis with Annexin-V-FITC staining, we identified significantly increased levels of programmed cell death for the combination of DCA and PX-478 in MCF-7 cells compared to PX-478-treated cells (Fig. 6). While 12% of PX-478-treated cells were Annexin-V-FITC-positive, the percentage increased to 20% after combination treatment (*p* = 0.004). Thus, we concluded that apoptosis is a relevant factor for this synergism in HT-29 and MCF-7 cells.

For both cell lines, pRB1 levels were significantly lower in combination-treated cells than in single compound-treated cells and control cells (Fig. 5). Furthermore, we observed an interesting effect of the combination in MCF-7 cells: while PX-478 alone did not affect the level of pRB1 at 0.5 x EC_50_ and EC_50_, DCA led to decreased levels of pRB1 (52 and 54%, respectively). For the combination, pRB1 levels were reduced to 33% compared to control at the lower concentration and 25% at the higher concentration (*p* = 0.027 and 0.046 compared to the single compounds, respectively). These data suggest that DCA alone has limited effects on pRB1 levels in MCF-7 cells while the combination affects RB1 phosphorylation more strongly.

Furthermore, we used Western blotting to evaluate the impact of the compounds on Cyclin D1 levels. In HT-29 and MCF-7 cells, the level of Cyclin D1 exhibited the greatest reduction for the combination treatment (*p* = 0.009 and *p* = 0.005, respectively, compared to control treatment). However, the differences with respect to each single compound were non-significant (Fig. 5). Collectively, these data suggest that the combination of DCA and PX-478 synergistically reduces cell proliferation.

### The effect of DCA was verified via real-time measurement of metabolism (seahorse XFe96)

**Fig. 7:**
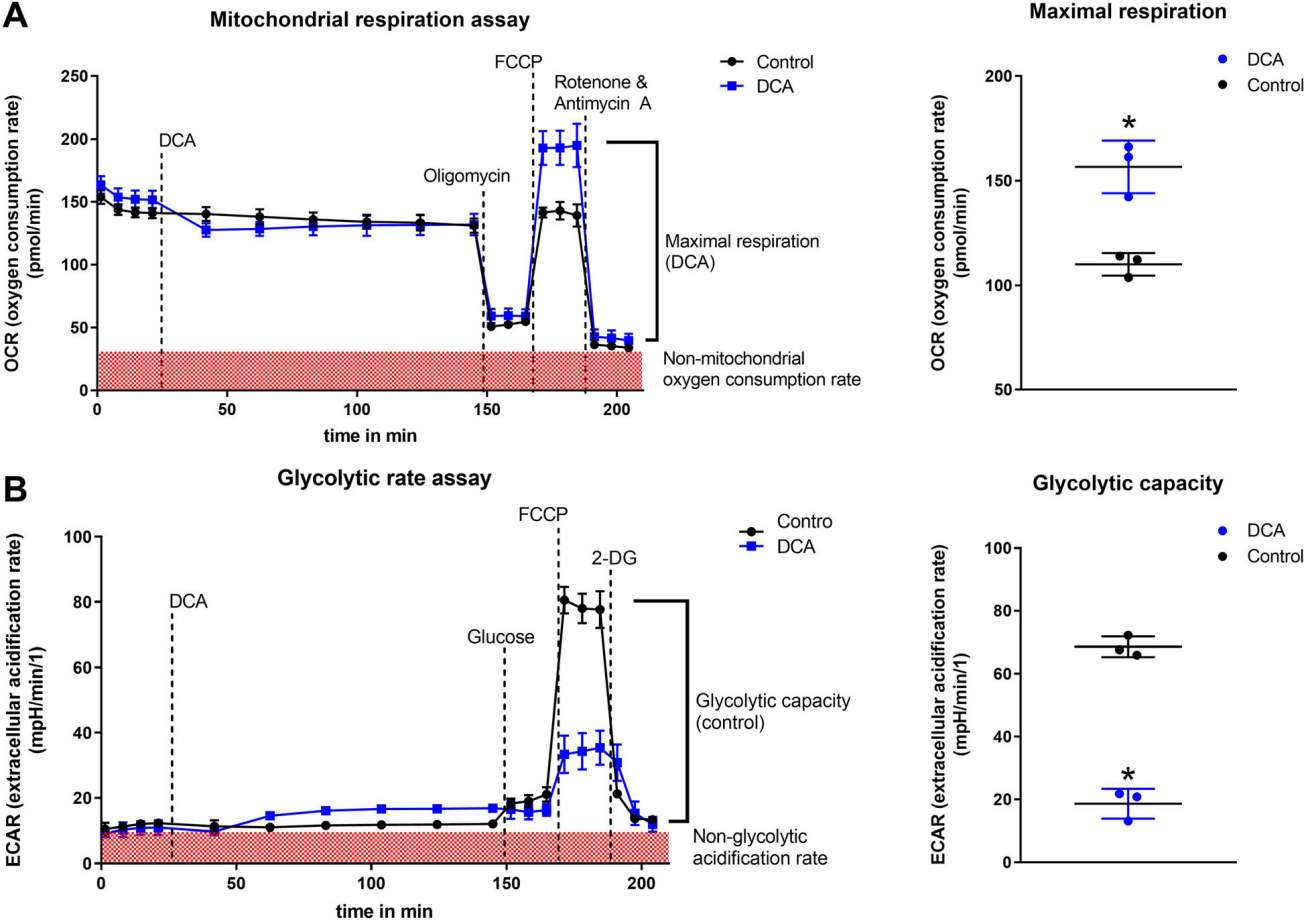
Mitochondrial respiration assay and glycolytic stress test with DCA. Figure 7 shows the results of the mitochondrial respiration assay (**a**) and glycolytic stress test (**b**) of DCA-treated MCF-7 cells performed with a Seahorse XFe96 analyzer. Three independent experiments were performed with at least four technical replicates. After measurement of the baseline OCR and ECAR, the EC_50_ dose of DCA was injected. After six measurement cycles, oligomycin (inhibition of ATP synthase), FCCP (uncoupling agent) and rotenone & antimycin A (inhibition of respiratory chain) were added for the mitochondrial respiration assay (**a**), and glucose, oligomycin and 2-DG (inhibition of glycolysis) were added for the glycolytic rate assay (**b**). Three measurement cycles were performed after each chemical was added. **a:** As shown, DCA increased the maximal OCR and thereby the maximal respiration of MCF-7 cells. **b:** Furthermore, DCA reduced the maximal ECAR and thereby the glycolytic capacity. See the Methods section for the exact calculation procedures and definitions of glycolytic capacity, maximal respiration, non-mitochondrial OCR and non-glycolytic acidification rate. In MCF-7 cells, DCA increased the maximal respiration by 42% and decreased the glycolytic capacity by 73% (*p* = 0.004 and 0.0001, respectively)

To verify the effects of DCA on glycolysis, studies with the Seahorse XFe96 Analyzer were performed (Fig. 7). We measured real-time changes in the oxygen consumption rate (OCR) and the extracellular acidification rate (ECAR). Two hours after treatment with DCA, the protocols for the mitochondrial respiration assay and the glycolytic rate assay were performed. The results supported the hypothesis that DCA increases the influx of pyruvate into mitochondria, which led to a 42% increase in maximal respiration (*p* = 0.004). In addition, we observed a 73% reduction in glycolytic capacity when DCA was added (*p* = 0.0001).

## Discussion

In this study, we demonstrate that DCA and PX-478 are a potent combination that exerts synergistic effects in all tested cancer cell lines and proved thereby to be effective in various tumor entities in vitro including colorectal, lung, breast, cervical, liver and brain cancer while having limited effects on the non-cancerous cell line HEK-293 (Figs. 1 and 2). We found the combination to induce cell cycle arrest and apoptosis as well as increasing the generation of ROS in a colorectal and a breast cancer cell line (HT-29 and MCF-7).

The EC_50_ of PX-478 ranged from 11.2 to 276 μM, indicating a drug resistance for MDA MB-231 cells (276 μM). Interestingly, this resistance does not inhibit synergism, with a CI value of 0.8. However, best CI values where lower in all other cell lines. Via its effect on HIF-1α, PX-478 has already shown synergistic potential with different compounds. In combination with arsenic trioxide (ATO), PX-478 increases ROS and, likely, ROS-induced apoptosis [24]. As our data suggest, this mechanism might also apply to the combination of DCA + PX-478. Interestingly, both DCA and PX-478 mediate antitumoral effects through inhibition of PDKs, which can partially explain the synergism observed here. While DCA suppresses PDK-1, HIF-1α increases PDK-1 expression [27, 28]. Thus, PX-478 reinforces the primary effect of DCA indirectly, thereby synergistically increasing ROS production when combined with DCA, as our data suggest (Figs. 3 and 4). These results are in line with the findings of Lang et al. and support the hypothesis that PX-478, as a HIF-1α inhibitor, may be beneficial for different therapeutic approaches.

The EC_50_ of DCA ranged from 21.2 mM to 41.9 mM (Fig. 2). A heterogeneity of the DCA-mediated effects in different cancer cell lines can be seen when our real-time metabolic assay results are compared with those of Tataranni et al. and Lucido et al. [17, 33]. DCA strongly increased maximal respiration and decreased glycolytic capacity in MCF-7 cells (Fig. 7), while in pancreatic carcinoma as well as head and neck squamous cell cancer, both glycolytic capacity and maximal respiration were decreased. Consistent with our findings however, Ma et al. found increased maximal respiration in non-small cell lung cancer cells treated with DCA [34].

Hence, literature as well as our data suggest that DCA mediates heterogenic metabolic modulation depending on the metabolic status of a cancer cell. Interestingly, cells primarily undergoing oxidative phosphorylation as well as cells relying primarily on aerobic glycolysis can both be sensitive to DCA [35–39].

As DCA has attracted considerable attention in recent years, many examples of synergism have been detected. 5-Fluorouracil, a platinum-based chemotherapy, a SIRT2 inhibitor, metformin, omeprazole + tamoxifen, sorafenib, erlotinib and gefitinib have shown synergistic effects in combination with DCA in vivo and in vitro [15, 34, 40–48].

Clinical trials with DCA in cancer therapy, congenital lactic acidosis and pulmonary arterial hypertension have been performed in recent decades and are ongoing [49–51]. Although DCA has not yet been implemented in clinical cancer treatment regimens, interest in DCA has not decreased. Authors of clinical trials with DCA suggest DCA in combination with chemotherapy in previously treated metastatic breast cancer and non-small cell lung cancer (ClinicalTrials.gov Identifier: NCT01029925) [45] and as an apoptosis sensitiser for recurrent solid tumors (ClinicalTrials.gov Identifier: NCT00566410) in less advanced disease stage [52].

Although a phase 1 clinical trial of PX-478 conducted in 2010 in patients with advanced solid tumors showed that PX-478 was well tolerated at low doses, with consistent HIF-1α inhibition and prolonged duration of stable disease [53], it seems to have been abandoned as an anticancer drug, as no further clinical trials with PX-478 have been registered. If PX-478 is used in combination with DCA, obstacles such as its dose-limiting toxicity could be eliminated. We believe that synergism is an important strategy for successfully including promising compounds such as DCA and/or PX-478 in cancer therapy. Our data indicates that the concentrations of DCA and PX-478 could be reduced by an average of approximately 60.7%. Considering the concentrations of DCA achieved in clinical studies and our EC_50_ values in the different cell lines tested, we conclude that combination of DCA and PX-478 can help attain the concentrations needed for a therapeutic effect.

### Limitations

In this study, we focused on the effect of the specific compounds and their combination rather than identifying whether a certain effect can be directly linked to a specific mode of action of a single compound. These conclusions must be drawn considering the existing data for single compounds.

While DCA exerts an immediate effect via PDH activation (see the results of the real-time metabolic assays, Fig. 7), PX-478-mediated inhibition of the transcription factor HIF-1α consequently shows relatively delayed effects. HIF-1α, having a short half-life of eight to 20 min itself [54], regulates more than 100 proteins, exemplarily GLUT1 and VEGFA, with half-lifes of approximately 7–8 h [55, 56].

We performed Western blot analysis after 24 or 48 h of incubation to partially address this issue, but we did not consistently quantify the individual effects of DCA and PX-478 at the respective time points. Consequently, we did not analyse the dynamics of this combination.

### Conclusion

In summary, we found synergistic effects of the combination DCA and PX-478 in all analysed cancer cell lines, including colorectal, lung, breast, cervical, liver and brain cancer. Induction of apoptosis, generation of ROS and inhibition of proliferation played important roles in this synergism. Considering the promising synergism between the two compounds presented here and the evidence generated by various research groups about the effects of DCA and PX-478, commencement of in vivo trials (e.g. xenografts) is recommended.

## Supplementary Information

**Additional file 1.** Includes data of: Combination experiments with DCA and PX-478, flow cytometric analysis, HPLC analysis, Western blot analysis and Seahorse analysis.

**Additional file 2.** Includes all Western Blots.

## Data Availability

All data generated or analysed during this study are included in this published article and its supplementary files (additional file 1 and additional file 2).

## Abbreviations

CI: Combination index
DCA: Dichloroacetate
FACS: Fluorescence-activated cell sorting
HPLC: High performance liquid chromatography
HIF-1α: Hypoxia inducible factor α
ROS: Reactive oxygen species
w/v: Weight per volume
v/v: Volume per volume
