# Supplementary material for: Dichloroacetate and PX-478 exhibit strong synergistic effects in a various number of cancer cell lines
Source: BMC Cancer. 2021 Apr 30;21:481. doi: 10.1186/s12885-021-08186-9 (PMC8086110; doi:10.1186/s12885-021-08186-9)
Supplement: Supplementary file 2 — Additional file 2. Includes all Western Blots. [file 12885_2021_8186_MOESM2_ESM.pptx]

## Slide 1
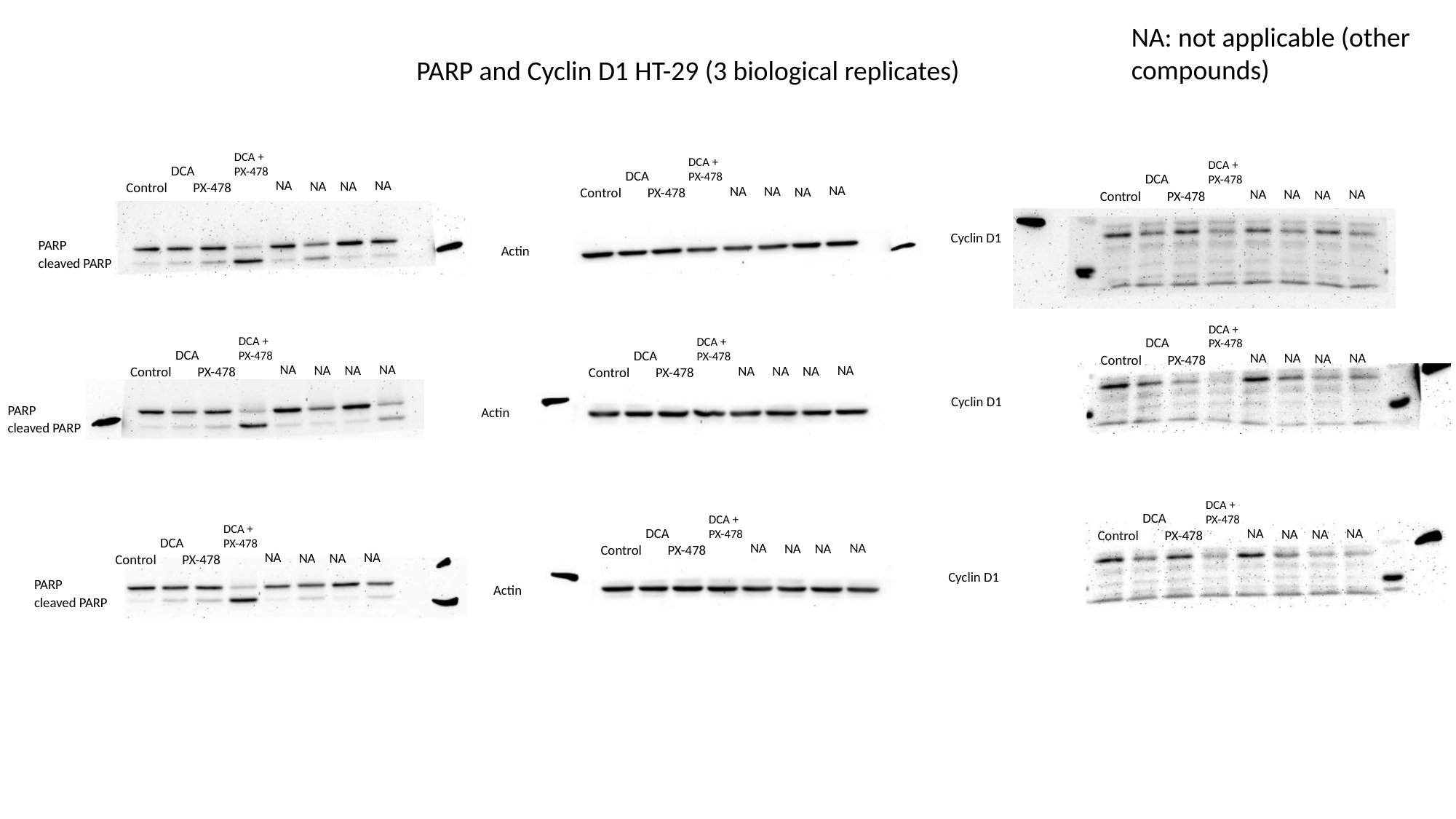

NA: not applicable (other compounds)
PARP and Cyclin D1 HT-29 (3 biological replicates)
DCA + PX-478
DCA + PX-478
DCA + PX-478
DCA
DCA
DCA
NA
NA
NA
NA
Control
PX-478
NA
NA
NA
NA
Control
PX-478
NA
NA
NA
NA
Control
PX-478
Cyclin D1
PARP
Actin
cleaved PARP
DCA + PX-478
DCA + PX-478
DCA
DCA + PX-478
DCA
DCA
NA
NA
NA
NA
Control
PX-478
NA
NA
NA
NA
NA
NA
NA
NA
Control
PX-478
Control
PX-478
Cyclin D1
PARP
Actin
cleaved PARP
DCA + PX-478
DCA
DCA + PX-478
DCA + PX-478
DCA
NA
NA
NA
NA
Control
PX-478
DCA
NA
NA
NA
NA
Control
PX-478
NA
NA
NA
NA
Control
PX-478
Cyclin D1
PARP
Actin
cleaved PARP

## Slide 2
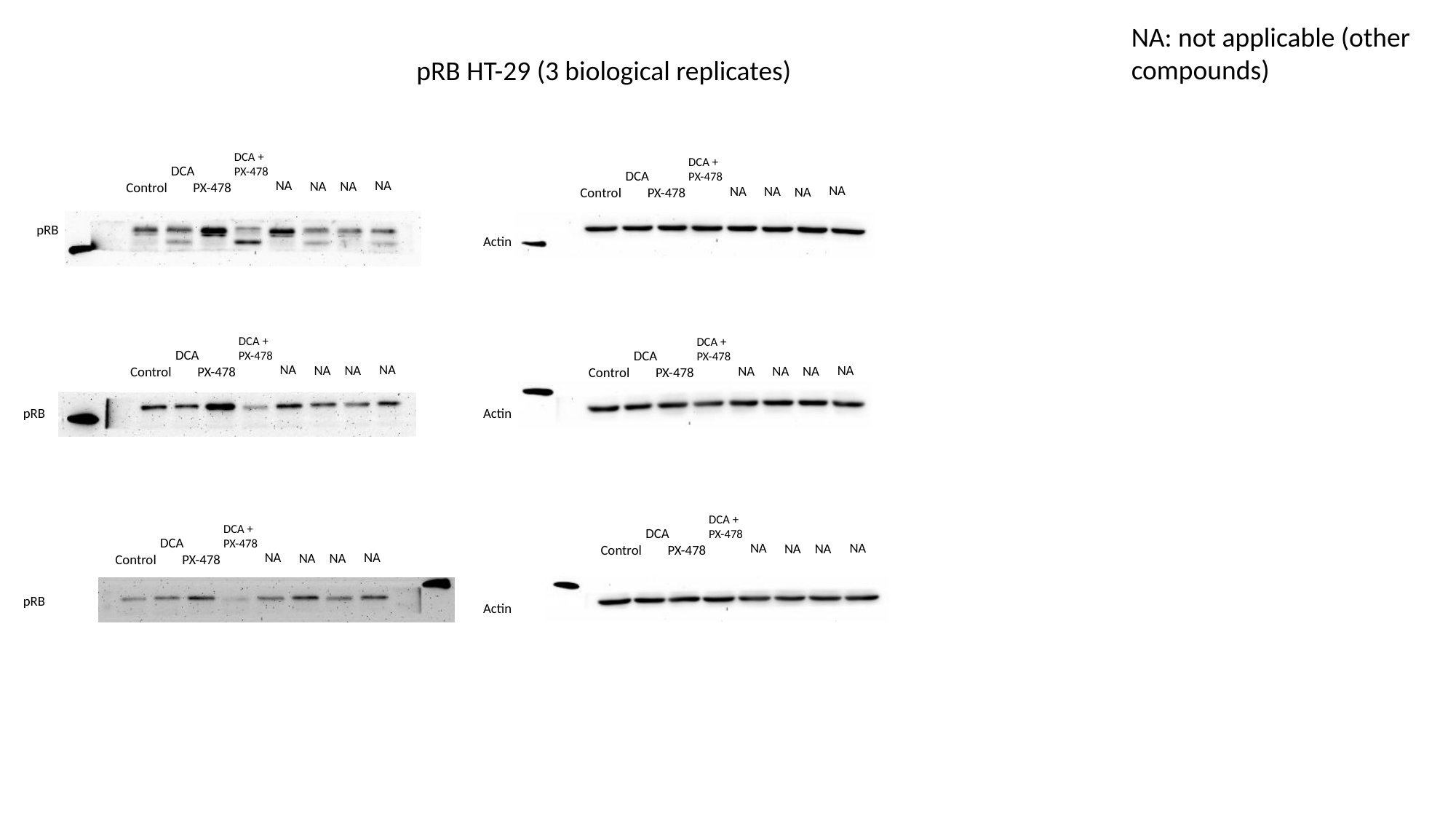

NA: not applicable (other compounds)
pRB HT-29 (3 biological replicates)
DCA + PX-478
DCA + PX-478
DCA
DCA
NA
NA
NA
NA
Control
PX-478
NA
NA
NA
NA
Control
PX-478
pRB
Actin
DCA + PX-478
DCA + PX-478
DCA
DCA
NA
NA
NA
NA
NA
NA
NA
NA
Control
PX-478
Control
PX-478
pRB
Actin
DCA + PX-478
DCA + PX-478
DCA
DCA
NA
NA
NA
NA
Control
PX-478
NA
NA
NA
NA
Control
PX-478
pRB
Actin

## Slide 3
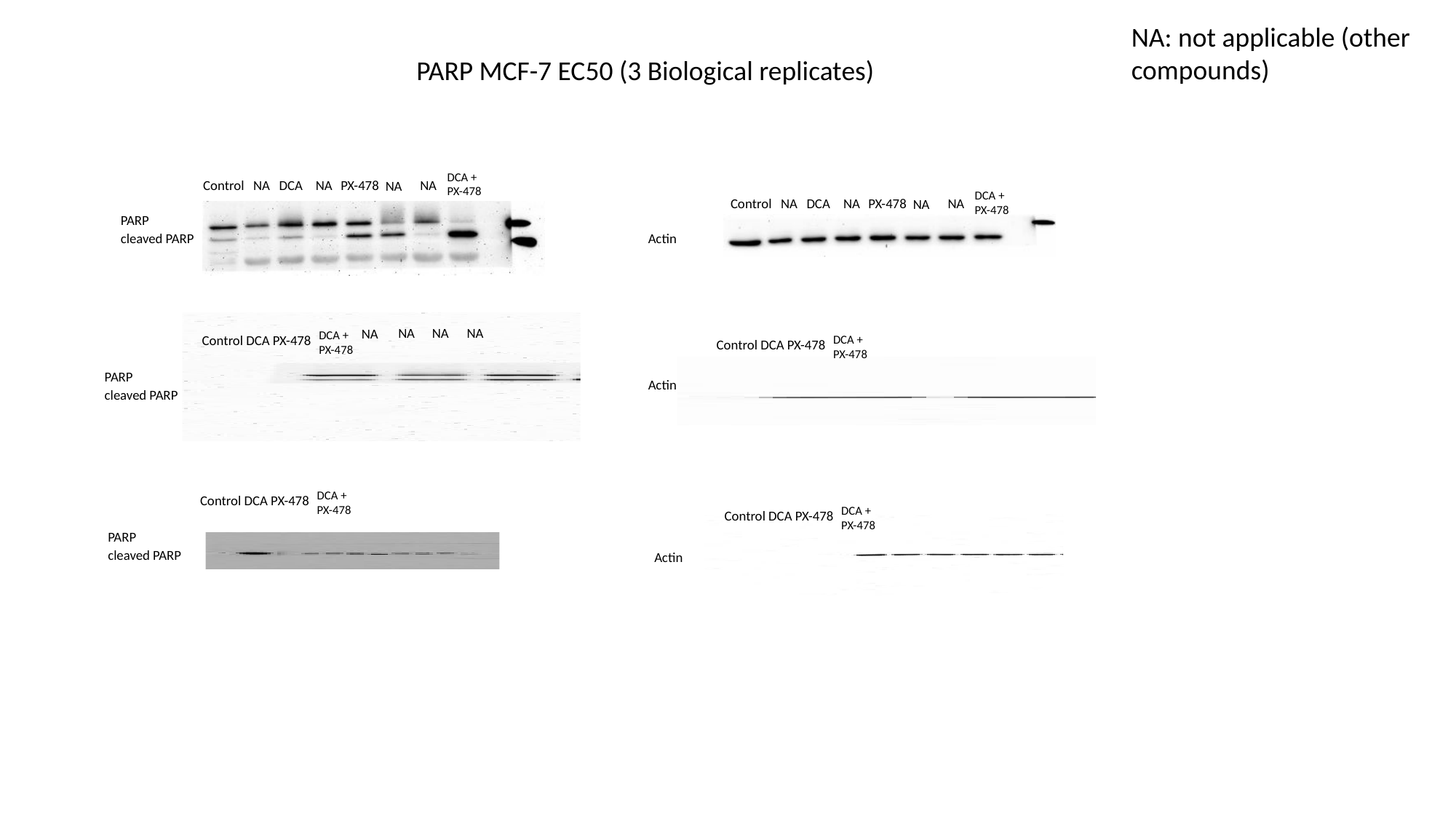

NA: not applicable (other compounds)
PARP MCF-7 EC50 (3 Biological replicates)
DCA + PX-478
NA
Control
NA
DCA
NA
PX-478
NA
DCA + PX-478
NA
Control
NA
DCA
NA
PX-478
NA
PARP
cleaved PARP
Actin
NA
NA
NA
NA
DCA + PX-478
Control DCA PX-478
DCA + PX-478
Control DCA PX-478
PARP
Actin
cleaved PARP
DCA + PX-478
Control DCA PX-478
DCA + PX-478
Control DCA PX-478
PARP
cleaved PARP
Actin

## Slide 4
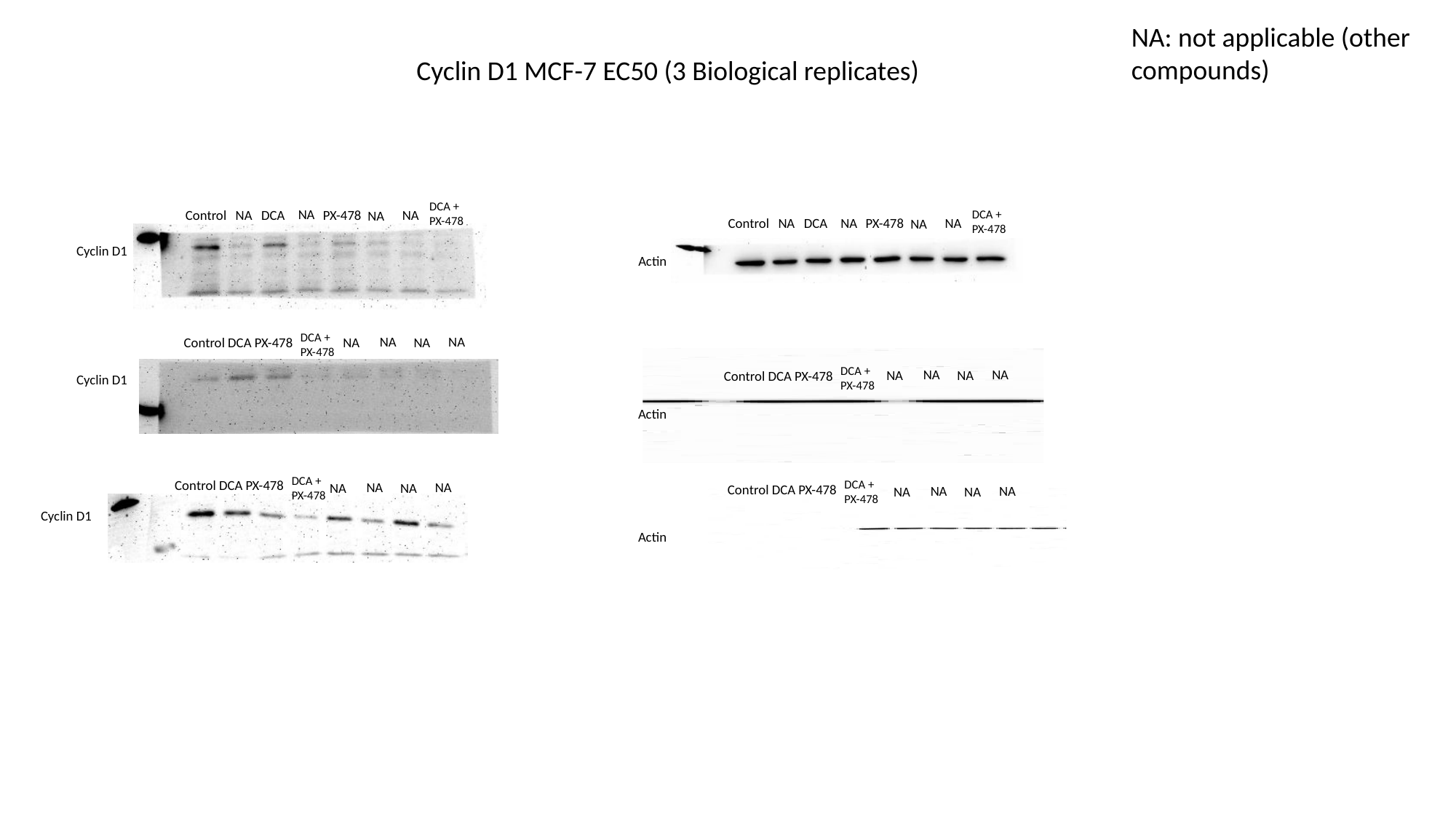

NA: not applicable (other compounds)
Cyclin D1 MCF-7 EC50 (3 Biological replicates)
DCA + PX-478
NA
Control
NA
DCA
NA
PX-478
NA
DCA + PX-478
NA
Control
NA
DCA
NA
PX-478
NA
Cyclin D1
Actin
DCA + PX-478
NA
NA
NA
Control DCA PX-478
NA
DCA + PX-478
NA
NA
NA
NA
Control DCA PX-478
Cyclin D1
Actin
DCA + PX-478
Control DCA PX-478
DCA + PX-478
NA
NA
NA
NA
Control DCA PX-478
NA
NA
NA
NA
Cyclin D1
Actin

## Slide 5
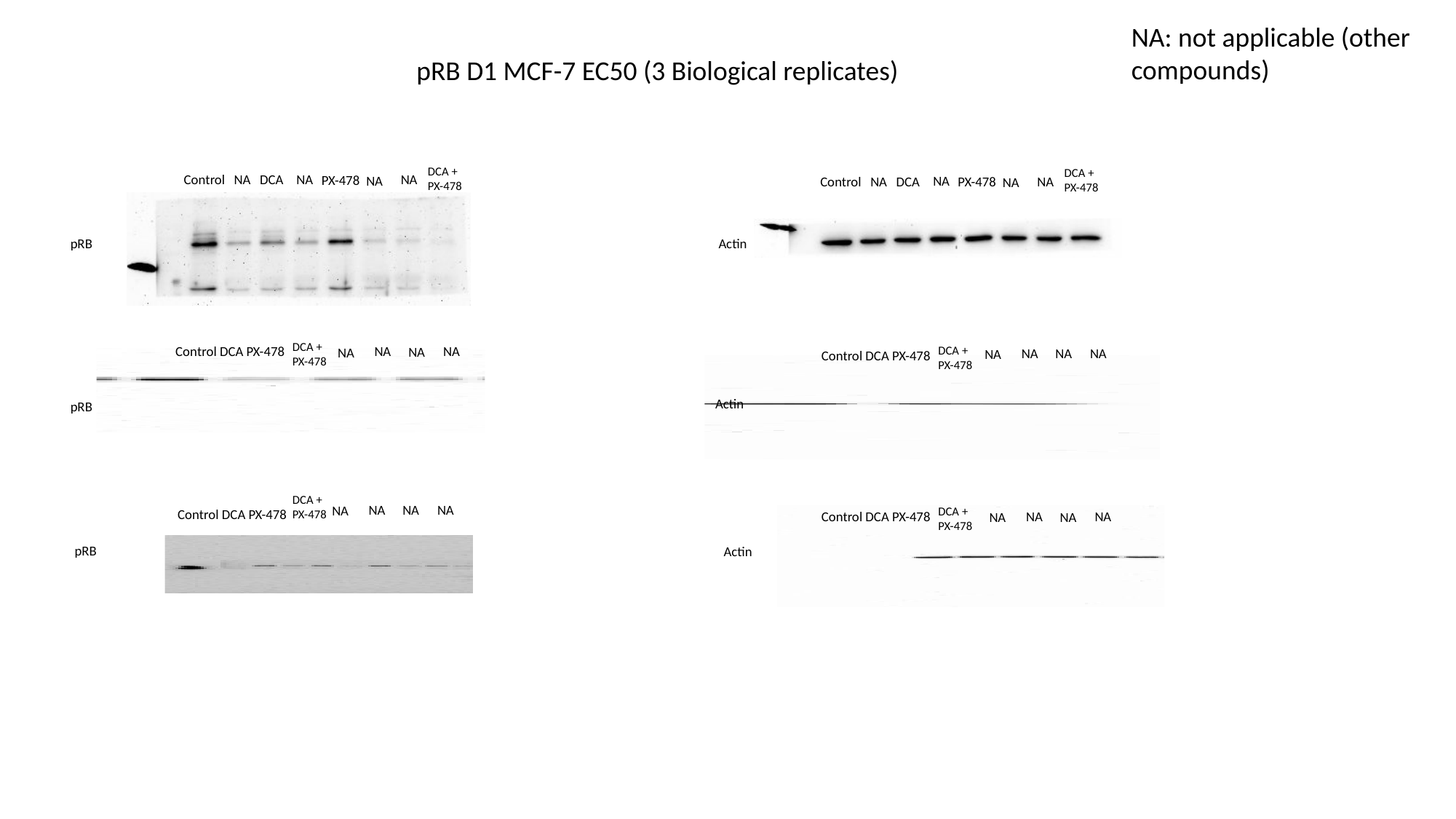

NA: not applicable (other compounds)
pRB D1 MCF-7 EC50 (3 Biological replicates)
DCA + PX-478
DCA + PX-478
NA
Control
NA
DCA
NA
PX-478
NA
NA
Control
NA
DCA
NA
PX-478
NA
pRB
Actin
DCA + PX-478
Control DCA PX-478
NA
NA
DCA + PX-478
NA
NA
NA
NA
NA
NA
Control DCA PX-478
Actin
pRB
DCA + PX-478
NA
NA
NA
NA
DCA + PX-478
Control DCA PX-478
Control DCA PX-478
NA
NA
NA
NA
pRB
Actin

## Slide 6
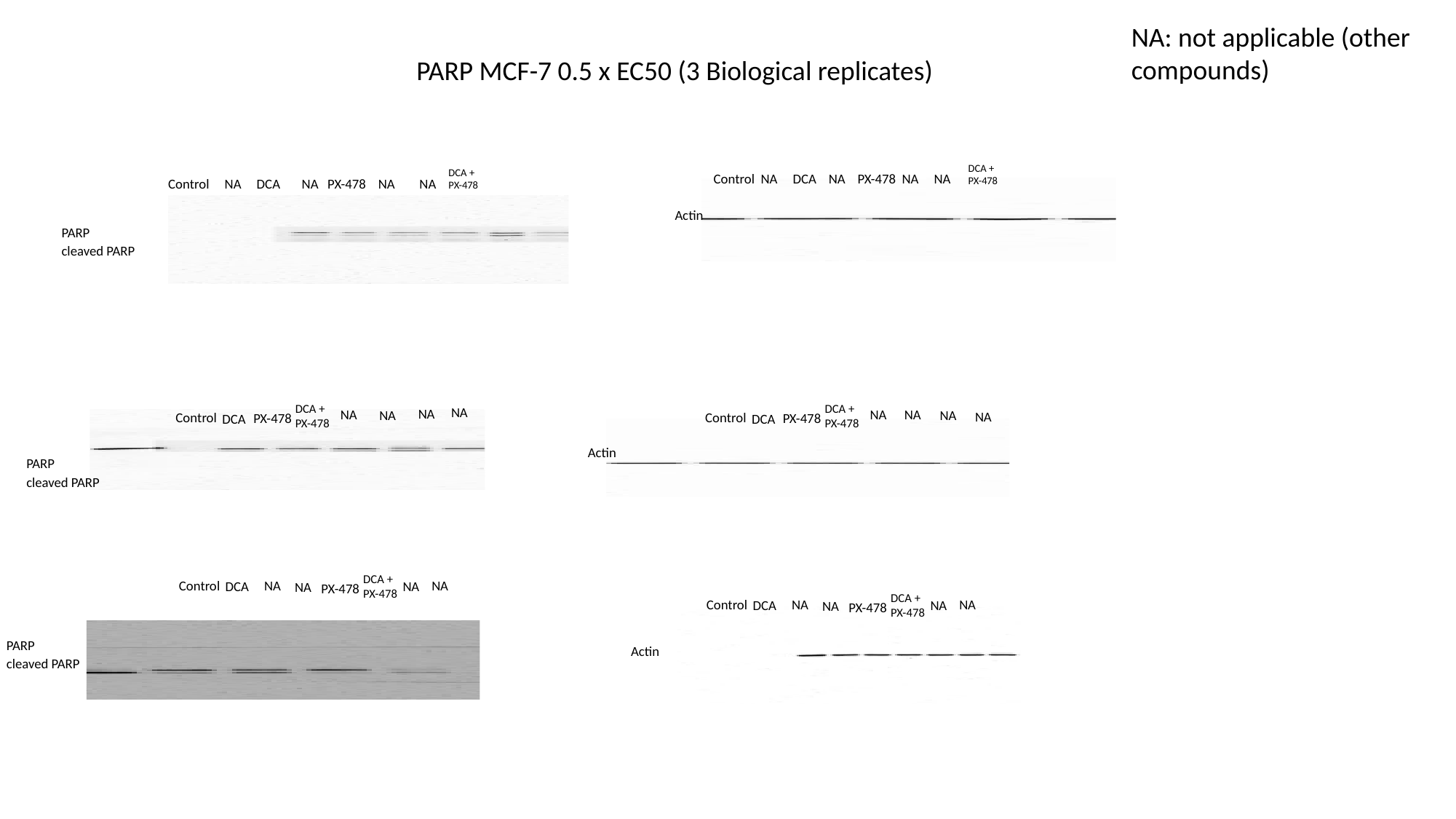

NA: not applicable (other compounds)
PARP MCF-7 0.5 x EC50 (3 Biological replicates)
DCA + PX-478
DCA + PX-478
Control NA DCA NA PX-478 NA NA
Control NA DCA NA PX-478 NA NA
Actin
PARP
cleaved PARP
DCA + PX-478
DCA + PX-478
NA
NA
NA
NA
NA
NA
NA
NA
Control
Control
PX-478
PX-478
DCA
DCA
Actin
PARP
cleaved PARP
DCA + PX-478
Control
NA
NA
DCA
NA
NA
PX-478
DCA + PX-478
Control
NA
NA
DCA
NA
NA
PX-478
PARP
Actin
cleaved PARP

## Slide 7
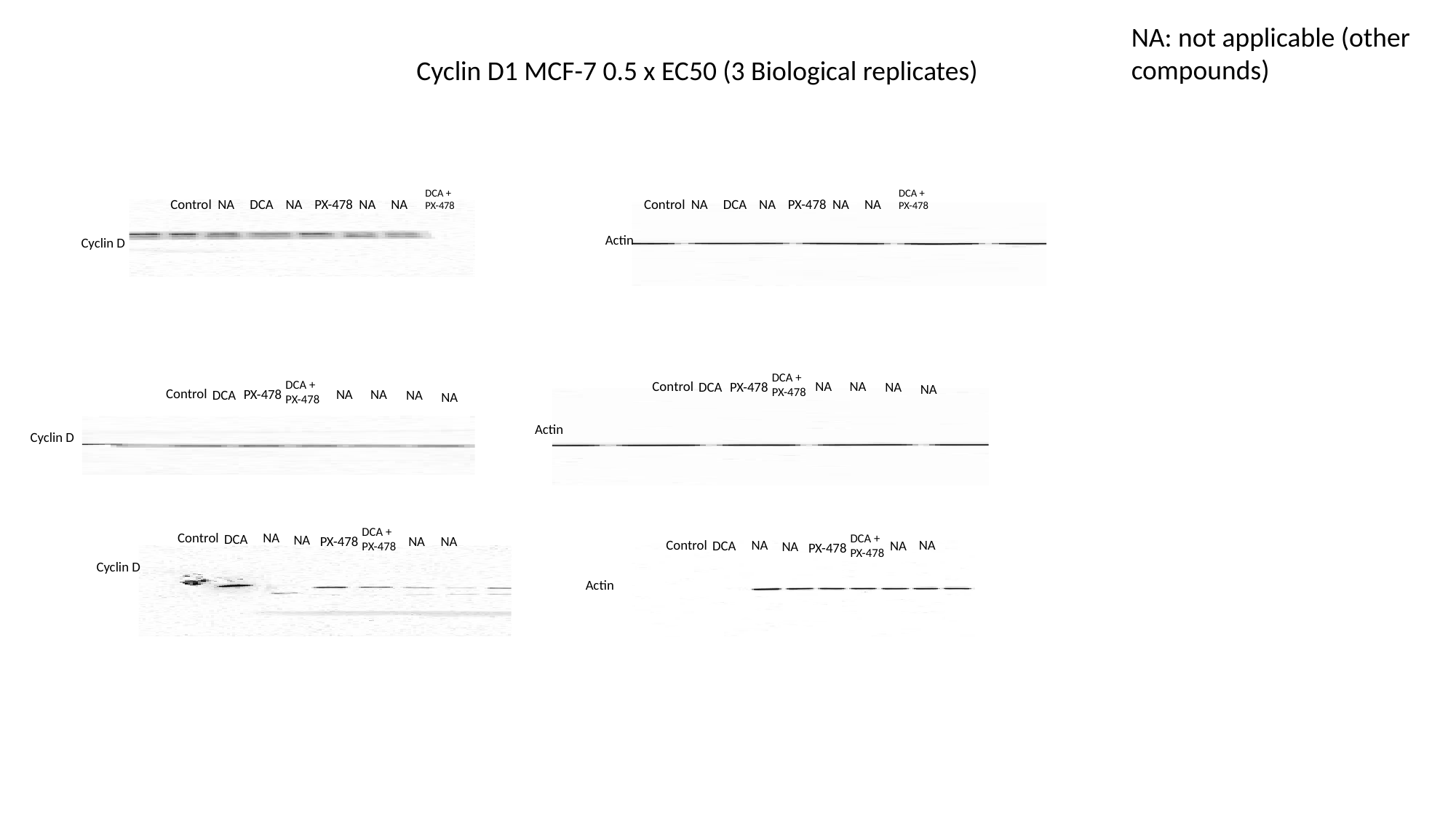

NA: not applicable (other compounds)
Cyclin D1 MCF-7 0.5 x EC50 (3 Biological replicates)
DCA + PX-478
DCA + PX-478
Control NA DCA NA PX-478 NA NA
Control NA DCA NA PX-478 NA NA
Actin
Cyclin D
DCA + PX-478
Control
DCA + PX-478
NA
NA
PX-478
NA
DCA
NA
Control
NA
NA
PX-478
DCA
NA
NA
Actin
Cyclin D
DCA + PX-478
Control
NA
DCA
DCA + PX-478
NA
PX-478
NA
NA
Control
NA
NA
DCA
NA
NA
PX-478
Cyclin D
Actin

## Slide 8
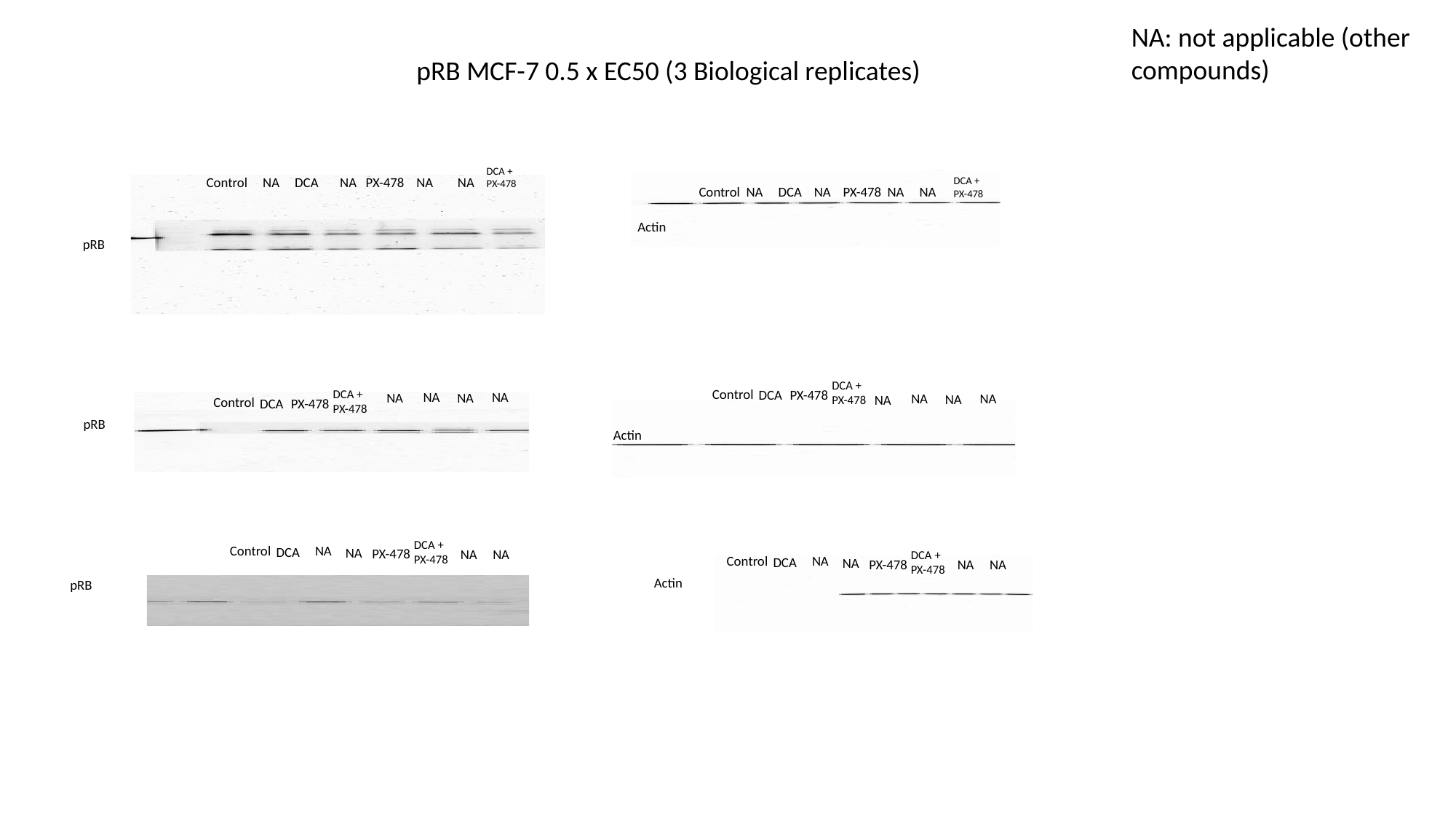

NA: not applicable (other compounds)
pRB MCF-7 0.5 x EC50 (3 Biological replicates)
DCA + PX-478
Control NA DCA NA PX-478 NA NA
DCA + PX-478
Control NA DCA NA PX-478 NA NA
Actin
pRB
DCA + PX-478
Control
DCA + PX-478
PX-478
DCA
NA
NA
NA
NA
NA
NA
NA
NA
Control
PX-478
DCA
pRB
Actin
DCA + PX-478
Control
NA
DCA
NA
PX-478
NA
NA
DCA + PX-478
Control
NA
DCA
NA
PX-478
NA
NA
Actin
pRB
